# Machine learning prediction of long-term sickness absence due to mental disorders using Brief Job Stress Questionnaire data

**DOI:** 10.1038/s41598-025-32857-3

**Published:** 2025-12-16

**Authors:** Shinichi Iwasaki, Yasuhiko Deguchi, Shohei Okura, Kunio Maekubo, Ayaka Matsunaga, Koki Inoue

**Affiliations:** Department of Neuropsychiatry, Graduate School of Medicine, Osaka Metropolitan University, 1-4-3, Asahimachi, Abeno-ku, Osaka, 545-8585 Japan

**Keywords:** Long-term sickness absence, Mental disorder, Brief Job Stress Questionnaire, Stress check system, Machine learning, Sampling method, Imbalanced data, Psychiatric disorders, Occupational health

## Abstract

Long-term sickness absence (LTSA) is a significant issue, causing productivity decline, financial difficulties, and increased mental health issues, with mental disorders being the most common cause. Occupational stressors are also linked to increased risk of LTSA due to mental disorders (LTSA-MD). This study uses occupational stressors data, assessed using the Brief Job Stress Questionnaire from 2011 to 2022, to predict LTSA-MD using machine learning and sampling methods, assessing their performance. This study analyzes data from 231,425 Japanese public servants from 2011 to 2022, focusing on LTSA-MD incidents. We compared five machine learning models and six sampling methods (random sampling, equal size sampling, SMOTE-synthetic minority oversampling technique, bootstrapping, borderline-SMOTE and ADASYN-adaptive synthetic sampling) to predict LTSA-MD incidents, addressing class imbalance. We prioritized average precision (AP) to identify the most promising model–sampling combinations to give the severe class imbalance. The gradient boosted trees model and bootstrap oversampling method demonstrated highest AP among all integrations of machine learning and sampling methods, with a AP of 0.040 and a ROC-AUC of 0.81. However, no significant difference in superiority was observed between the combinations of higher-level AP machine learning and sampling methods. The results demonstrate that machine learning models’ predictive ability for LTSA-MD is generally low, requiring further research.

**Supplementary Information:**

The online version contains supplementary material available at 10.1038/s41598-025-32857-3.

## Introduction

Long-term sickness absence (LTSA) induces productivity decline and additional costs related to sick pay, hiring temporary replacements in the workplace, financial difficulties due to reduced income, increased medical expenses, feelings of isolation, decreased self-esteem, and exacerbation of mental health issues in an individual’s career and later life^1^. The main causes of LTSA are mental disorders and chronic physical conditions^2,3^. Mental disorders are especially a significant reason for LTSA^4^. From a longitudinal perspective, a change in mental health influences absenteeism more than three times greater than a change in physical health^5^. This study shows that mental disorders were by far the most common reason for LTSA^3^^,^ and the average length of long-term leave due to all mental illnesses was 13.2 months among Japanese workers, which had a significantly long negative impact on the workplace. Therefore, predicting LTSA due to mental disorders (LTSA-MD) is crucial for developing effective prevention strategies.

However, occupational stressors were also positively associated with an increased risk of all sick leave^6^. In a meta-analysis, workers exposed to occupational stressors were at a 76% higher risk of sickness absence due to mental disorders than those not exposed^7^. LTSA-MD is associated with high job strain, low social support at work, effort-reward imbalance, and high overcommitment to work^8^.

Therefore, predicting long-term absences from work due to mental illness based on occupational stressors experienced by workers can be very useful for building efficient intervention methods. According to several studies, LTSA and LTSA-MD are predicted by individual or combined workplace stressors or sociodemographic factors such as age, gender, education, symptoms of depression, anxiety, somatization, recovery expectations, and diversity of employment^9–11^. However, previous studies on LTSA-MD have several notable limitations. First, most prior investigations have been restricted by small sample sizes and limited follow-up periods, which reduces the reliability of their findings. Second, many studies have relied on conventional statistical approaches that do not adequately handle the severe class imbalance inherent in LTSA-MD, where the incidence is extremely low. As a result, models often default to predicting the absence of LTSA-MD, leading to poor detection of true cases. Third, existing predictive models frequently suffer from a lack of external validity, as their performance has rarely been tested across different occupational groups or large-scale populations. These gaps demonstrate the importance of more robust approaches that leverage large-scale datasets and advanced analytical methods to evaluate the feasibility of predicting LTSA-MD.

Recent studies have highlighted the potential of machine learning (ML) approaches for health-related prediction tasks, demonstrating their ability to capture complex nonlinear patterns^12–16^. Nonetheless, recent work has highlighted that several methodological and practical challenges remain in applying ML to the automated detection of mental illness, emphasizing the necessity of more rigorous and interpretable approaches^17^. In addition, the use of ML to predict mental disorders has increased^18^, and its applications have expanded across diverse health-related prediction tasks. For example, using a regression model and a decision tree model that equally well identified workers at risk of LTSA-MD^18^, and the decision tree model provides better insight into the LTSA-MD risk. However, their external validity study found that the predictive model and decision tree had poor ability to distinguish between participants with and without LTSA-MDs at the one-year follow-up^19^. A Japanese study showed that a random forest model could be used to predict sick leave among employees at a manufacturing plant in Japan and develop an action plan to promote employee health^20^.

This study focuses on predicting LTSA-MD among Japanese public servants using comprehensive national data to fill the gaps in existing literature. The Stress Check System, a national policy in Japan, is a standard occupational stress assessment method with the most common sample size. A few studies still use these stress check data to predict sickness absence due to mental illness using machine learning. Therefore, this study uses stress-check data from 2011 to 2022, and assesses the predictive ability of multiple ML methods for the incidence of LTSA-MD.

To address the aforementioned research gaps, the major contributions of this study are as follows: First, we utilize a large-scale, real-world dataset of 231,425 Japanese public servants to predict the rare event of LTSA-MD. Second, we systematically compare the performance of five machine learning models combined with four different sampling methods, providing a comprehensive evaluation of strategies to handle the severe class-imbalance problem inherent in LTSA-MD prediction. Third, by demonstrating the modest predictive power of models using only job stressor data, our study highlights the limitations of this approach and provides a clear direction for future research, such as the integration of clinical health data.

## Results

### Incidence of LTSA-MD

Table 1 presents the annual incidence of LTSA-MD among public servants from 2011 to 2022. Over the 12-year period, 1,216 workers experienced LTSA-MD, accounting for 0.34–0.84% of the workforce each year. The highest and lowest LTSA-MD rates were observed in 2022 (0.84%) and 2012, respectively (0.34%). The total number of workers yearly ranged from 18,002 to 21,266. The average number of days until the next Brief Job Stress Questionnaire (BJSQ) response was 372.3.

**Table 1:** Long-term sickness absence due to mental disorders incidences.

Year	LTSA-MD workers	Rate of LTSA-MD (%)	Total Number of Workers	Days to the next BJSQ (days)
2011	124	0.58%	21,266	442
2012	73	0.34%	21,198	319
2013	76	0.37%	20,293	331
2014	102	0.53%	19,390	365
2015	101	0.55%	18,479	383
2016	72	0.39%	18,257	366
2017	89	0.47%	18,760	364
2018	90	0.49%	18,264	364
2019	91	0.51%	18,002	371
2020	117	0.62%	18,848	357
2021	118	0.61%	19,247	365
2022	163	0.84%	19,421	440
Total	1,216		231,425	4,467

*LTSA-MD* long-term sickness absence due to mental disorders.

### Diagnosis of mental disorders for long-term sickness absence

Table 2 details the reasons for LTSA-MD classified by ICD-10 diagnostic codes^21^. The majority of cases were attributed to mood disorders (F3), accounting for 789 workers. This was followed by neurotic, stress-related, and somatoform disorders (F4), with 364 workers. Other categories included organic mental disorders (F0), mental and behavioral disorders due to psychoactive substance use (F1), and schizophrenia, schizotypal, and delusional disorders (F2), which affected 2, 24, and 27 workers, respectively. Less frequent diagnoses included behavioral syndromes associated with physiological disturbances and physical factors (F5), disorders of adult personality and behavior (F6), mental retardation (F8), and unspecified mental disorders (F9).

**Table 2:** Reason for long-term sickness absences due to mental disorders.

F0	2
F1	24
F2	27
F3	789
F4	364
F5	3
F6	1
F8	3
F9	3
Total	1,216

### Socio-demographic characteristics and BJSQ subscales

Table 3 summarizes the demographic variables and BJSQ subscale scores of the 231,425 participants, comparing males (64.5%) with females (35.5%). The average age was 45.1 years, with males being slightly older than females (46.3 vs. 42.9, respectively; *p* < 0.001). More males held managerial positions than women (64.4% vs. 35.6%; *p* < 0.001). Job type distribution showed males dominated technical roles, whereas females were more often professional or contract workers (*p* < 0.001). Significant sex differences were observed in BJSQ scores. Females reported higher quantitative and qualitative workloads, whereas males had higher physical demands and lower job control (*p* < 0.001). Females also reported more support from supervisors, coworkers, and family/friends, and higher support for physical stress responses (*p* < 0.001). Irritability and anxiety between men and women differed insignificantly.

**Table 3:** Demographic variables and Brief Job Stress Questionnaire subscale scores of participants.

Values		Range	Whole		Male		Female		*p*
Gender (n, %)			231,425		149,164	(64.5%)	82,261	(35.5%)	
Age		(20–65)	45.1 ± 10.2		46.3 ± 9.6		42.9 ± 10.8		***
Manager (n, %)			134,789	(58.2%)	86,864	(64.4%)	47,925	(35.6%)	
Job Types (n, %)									***
	Clerical workers		117,698	(50.9%)	83,196	(55.8%)	34,502	(41.9%)	
	Technical workers		51,407	(22.2%)	46,099	(30.9%)	5,308	(6.5%)	
	Professional workers		44,772	(19.3%)	13,798	(9.3%)	30,974	(37.7%)	
	Contract workers		17,548	(7.6%)	6,071	(4.1%)	11,477	(14.0%)	
Number of Department Member			63.4 ± 73.8		66.9 ± 69.0		57.0 ± 81.3		***
Department gender (male) composition			0.4 ± 0.3		0.2 ± 0.2		0.6 ± 0.3		***
BJSQ scores									
	Quantitative Workload	(3–12)	8.9 ± 2.8		8.7 ± 2.8		9.3 ± 2.8		***
	Qualitative Workload	(3–12)	9.2 ± 2.6		9.0 ± 2.6		9.6 ± 2.5		***
	Physical Demands	(1–4)	2.8 ± 1.0		2.9 ± 1.0		2.7 ± 1.1		***
	Low Job Control	(3–12)	6.8 ± 2.8		6.9 ± 2.8		6.7 ± 2.9		***
	Skill Underutilization	(1–4)	2.2 ± 0.8		2.3 ± 0.8		2.1 ± 0.9		***
	Interpersonal Conflict	(3–12)	6.1 ± 1.8		6.2 ± 1.8		6.0 ± 1.9		***
	Poor Physical Environment	(1–4)	2.4 ± 1.0		2.4 ± 1.0		2.4 ± 1.0		***
	Unsuitable Jobs	(1–4)	2.3 ± 0.8		2.3 ± 0.8		2.2 ± 0.8		***
	Low Meaningfulness of Work	(1–4)	2.2 ± 0.9		2.3 ± 0.8		2.1 ± 0.9		***
	Poor Vigor	(3–12)	9.2 ± 2.9		9.2 ± 2.9		9.2 ± 2.9		***
	Irritability	(3–12)	5.4 ± 2.8		5.4 ± 2.8		5.4 ± 2.8		
	Fatigue	(3–12)	5.8 ± 3.0		5.6 ± 2.9		6.2 ± 3.2		***
	Anxiety	(3–12)	5.3 ± 2.8		5.3 ± 2.7		5.2 ± 2.8		
	Depression	(6–24)	8.8 ± 4.5		8.9 ± 4.5		8.7 ± 4.5		***
	Physical Stress Responses	(11–44)	17.7 ± 8.0		17.4 ± 7.8		18.3 ± 8.3		***
	Low support from Supervisor	(3–12)	7.2 ± 2.3		7.2 ± 2.3		7.2 ± 2.4		***
	Low support from Coworker	(3–12)	6.8 ± 2.2		6.9 ± 2.2		6.6 ± 2.3		***
	Low support from Family/ Friends	(3–12)	5.2 ± 2.3		5.3 ± 2.3		4.9 ± 2.2		***
	Low Job Satisfaction	(2–8)	4.1 ± 1.4		4.2 ± 1.4		4.0 ± 1.3		***

Values are expressed by mean ± standard deviation.

Compared between gender, ***: p<0.001.

### Performance of machine learning algorithms and sampling methods

**Fig. 1:**
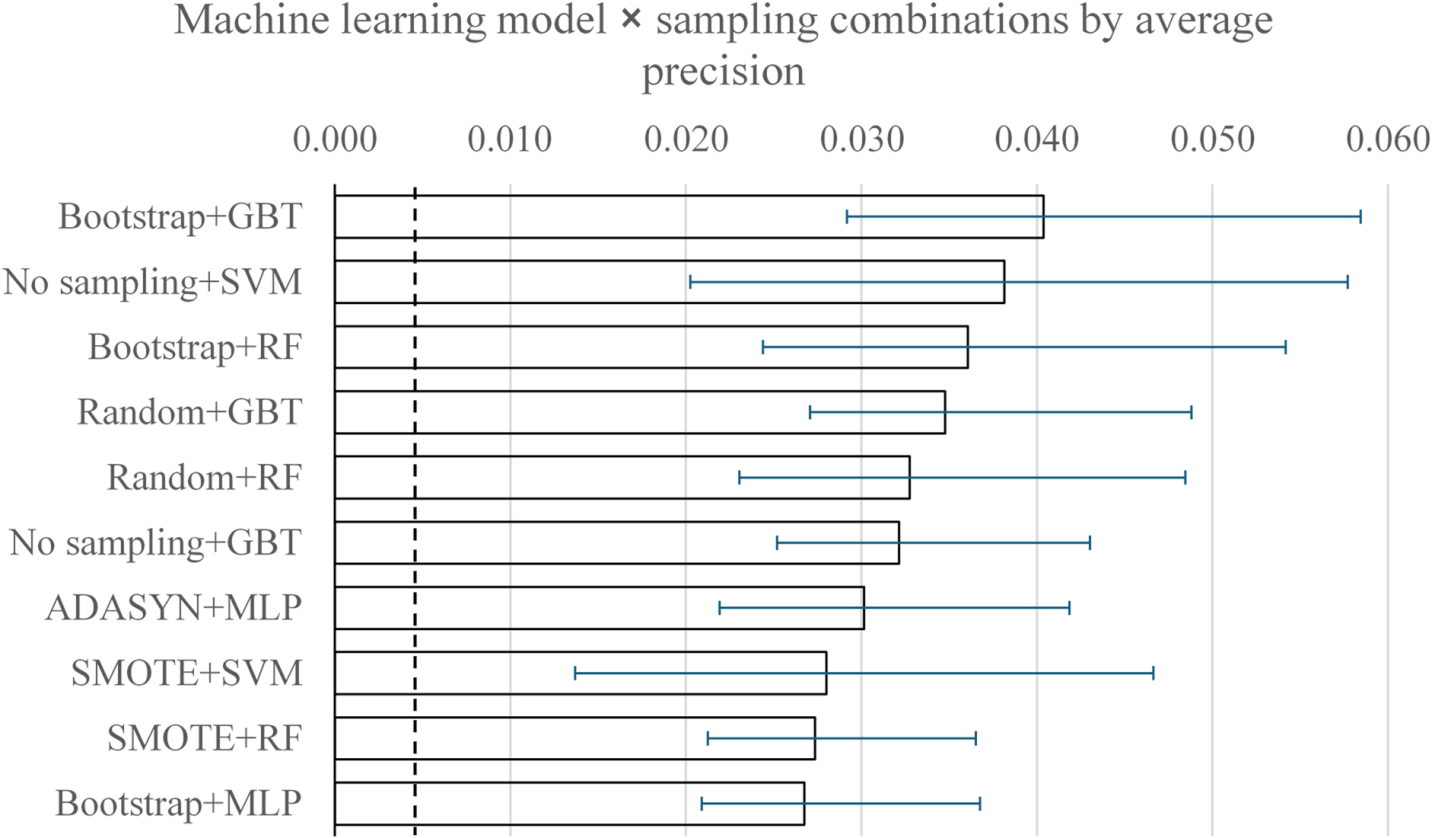
Average precision for the top-10 model–sampling. Average Precision (AP) for the AP top-10 model–sampling combinations with 95% bootstrapped CIs (percentile; B = 200). The dashed line indicates the baseline AP (= prevalence 0.0053). Algorithms: SVM: Support Vector Machine; RF: Random Forest; GBT: Gradient Boosted Trees; MLP: Multilayer Perceptron. Sampling: Bootstrap: Bootstrap 10 resamples; Random: 10% random sampling; SMOTE: Synthetic Minority Over-sampling Technique; ADASYN: Adaptive Synthetic Sampling.

Given the severe class imbalance, we used average precision (AP) as the primary precision-recall metric, which is consistent with stepwise interpolation; to identify the most promising model-sampling combinations. All combinations were evaluated on a common test set and ranked by AP; the top 10 are summarized in Fig. 1, a color-coded heatmap of AP for all algorithm × sampling combinations in the Supplementary Figure S1, and the full results are provided in Supplementary Table S1.

The top performers by AP were Bootstrap + Gradient Boosted Trees (GBT) (AP 0.040, 95% CI 0.029–0.058; ROC-AUC 0.806), No-sampling + SVM (0.038, 0.020–0.058; 0.503), and Bootstrap×10 + Random Forest (RF) (0.036, 0.024–0.054; 0.708). These values indicate modest discrimination in absolute terms, but an enrichment over the baseline AP (≈ 0.005) is expected from prevalence. Patterns across methods were consistent with rare-event learning: some heavy oversampling schemes increased recall substantially but at the cost of very low precision (reflected in low AP and MCC), whereas several no-sampling settings degenerated to near-all-negative predictions, yielding high accuracy yet low utility. For completeness, we also report ROC-AUC in Supplementary Table S2, noting its limited informativeness under a severe imbalance.

**Fig. 2:**
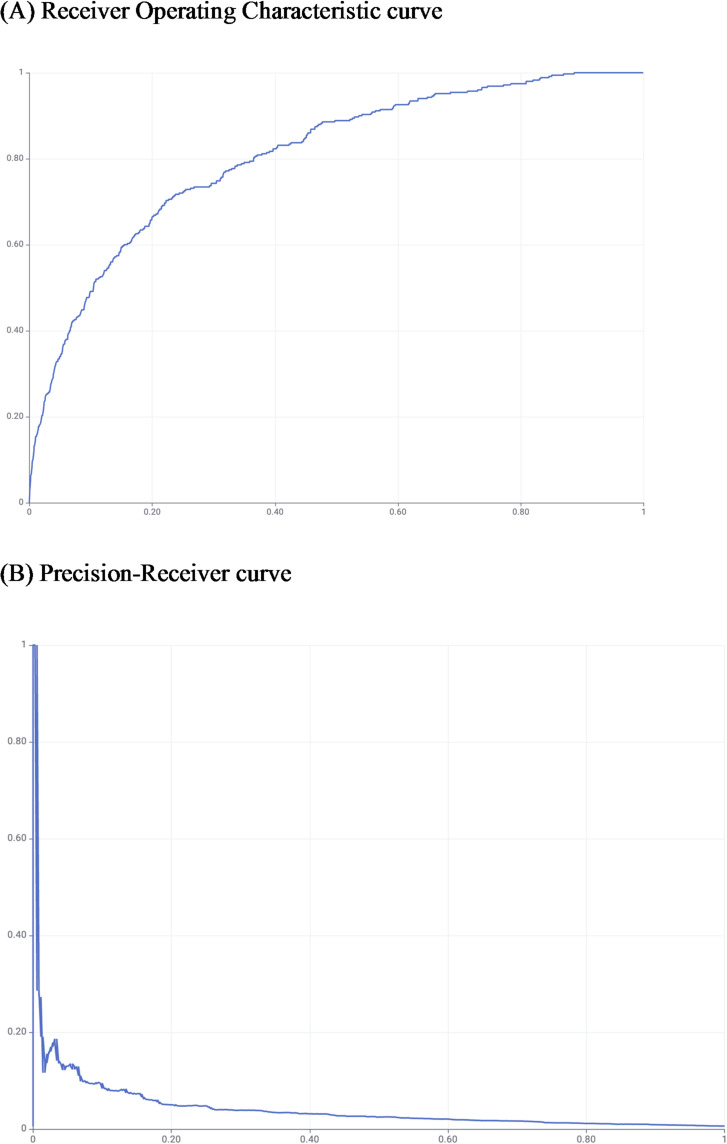
Receiver operating characteristic curve (**A**) and precision-receiver curve (**B**) for bootstrap×10 sampling and gradient boosted trees.

For formal model comparison, predictions were binarized by top-k (k equal to the number of positives in the test set, k = 350), and Cochran’s Q test was applied across the PR-AUC top-10 on identical cases (overall and, as sensitivity, positives-only). Cochran’s Q test showed no significant heterogeneity; therefore, no post-hoc McNemar tests were conducted per protocol. Although Cochran’s Q test did not detect an overall difference across the model–sampling combinations (Q = [5.36], df =^9^, p = [0.80]), we prespecified average precision as the primary metric given the severe class imbalance. On this basis, the bootstrap + gradient boosted trees model achieved the highest AP on the common test set and was considered the most promising combination for further reporting. Figure 2 displays the ROC (A) and PR (B) curves for gradient boosted trees trained with bootstrap resampling.

**Fig. 3:**
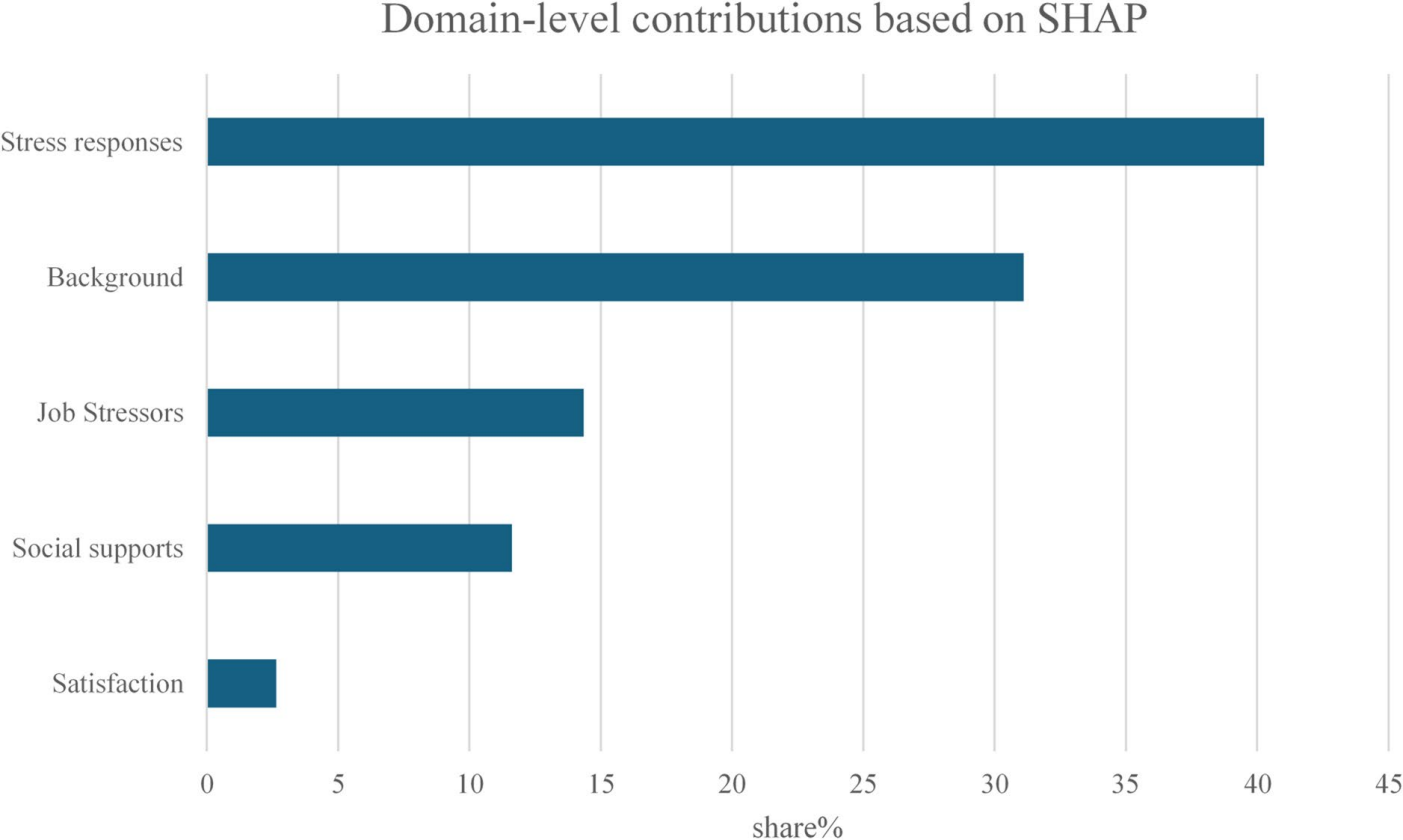
Domain-level contributions based on Shapley additive explanations (SHAP) of bootstrap and gradient boosted trees.

Table 4 lists the top features by mean |SHAP| for the top-ranked pipeline, including BJSQ item labels and question stems. Background factors—occupation, age, managerial status, and gender composition—appear alongside high-impact BJSQ items (e.g., worry/insecurity, workload, appetite loss, and vigor). At the domain level (Fig. 3), stress responses accounted for the largest share (40.27%), followed by background (31.11%), job stressors (14.35%), social supports (11.62%), and satisfaction (2.64%). These patterns are consistent with rare-event prediction on job-stressor data and align with the modest absolute discrimination reported above.

**Table 4:** Shapley additive explanations feature importance (top features by mean SHAP) for the top-ranked method (bootstrap and gradient boosted Trees) on the common test set.

Rank	Feature (code)	mean	share%	cum.%	Domain	BJSQ question
1	Occupation	0.38	9.07	9.07	Background	
2	Age	0.30	7.26	16.34	Background	
3	Manager	0.23	5.63	21.97	Background	
4	Gender composition	0.17	4.07	26.04	Background	
5	BJSQ-48	0.15	3.64	29.68	Social supports	“How freely can you talk with coworkers?”
6	Number of members	0.15	3.61	33.29	Background	
7	BJSQ-28	0.13	3.04	36.33	Stress responses	“I have felt worried or insecure.”
8	BJSQ-1	0.12	2.92	39.25	Job Stressors	“I have an extremely large amount of work to do.”
9	BJSQ-44	0.12	2.82	42.07	Stress responses	“I have lost my appetite.”
10	BJSQ-19	0.11	2.58	44.65	Stress responses	“I have been full of energy.”

*SHAP* Shapley additive explanations,* cum.* cumulative, *BJSQ* Brief Job Stress Questionnaire.

## Discussion

### Summary of findings

This study employs the most widely used BJSQ job stressor data from workers in Japan, and assesses the ability of multiple machine learning methods (logistic regression model, SVM, random forest, multilayer perceptron (MLP), and gradient boosted trees (GBT)) to predict the occurrence of LTSA-MD, using multiple sampling methods to handle the low incidence of LTSA-MD. The discriminative ability of LTSA-MD using BJSQ data was low for most of the machine learning and sampling methods used in this study.

The low predictive power may be attributable to the fact that the BJSQ data were too small to predict the incidence of LTSA-MD, or that they were of low relevance. Although we considered the possibility that the BJSQ score calculation would classify the BJSQ 57 items for better performance, the same analysis in this study was performed for the BJSQ subscale scores (Supplementary Table S3); however, no major differences were found between the results of this study and those of the BJSQ subscale scores. The BJSQ is designed as a simple questionnaire for use with a wide range of individuals, often featuring subscales with few items (1 to several questions). We anticipated that there would be little difference between analyzing individual questions and analyzing subscales, and the results confirmed this expectation.

Several studies have used the BJSQ to predict work absence, mainly using the Cox hazard model. In a study of workers in a financial services company (7356 male and 7362 female employees), the BJSQ was used to select high-stress individuals using the criteria recommended by the national stress check program, followed for one year. A Cox proportional regression model was further used to assess occupational stress and risk of sickness absence for one month or more. The hazard ratios for long-term absenteeism for those identified as high-stress individuals were 6.59 for men and 2.77 for women^22^. In another study, occupational stressors evaluated using the BJSQ among 197 healthy Japanese male and female workers, and physical demands were positively associated with an increased risk of sick leave in a Cox proportional hazards model (adjusted HR: 2.78)^6^. Higher job demands, as assessed by the BJSQ, were also found to be a risk factor (OR, 1.46) for recurrent sick leave due to depression after adjusting for confounding factors^23^. We also found an association between job stressors, stress responses, and job satisfaction scores on the BJSQ and an increased risk of LTSA-MD using data common to this study using the COX hazard model^24^. These studies indicate the predictability of sickness absence using BJSQ scores administered before the leave, and the use of the BJSQ to predict LTS-MD in this study was considered acceptable. However, the factors associated with sickness absence in these BJSQs varied by study, and were inconsistent. This may be largely due to characteristics such as the industry and role of the participants.

Several studies have used machine learning, similar to this study. A data analyzed by sparse logistic regression and a support vector machine for the risk of depression with questionnaires on general characteristics, physical health, job-related factors, psychosocial protective factors, and risk factors in the workplace among 503 employees^25^. The predictive accuracies of the three machine learning models using random forests were compared in terms of accuracy, precision, sensitivity, specificity, and the AUC of the Receiver Operating Characteristic (ROC) curve. All machine learning models showed similar results, with the random forests (88.7%) showing the highest accuracy. This study differs from the LTSA-MD because the outcome is depressive symptoms and a high CES-D score (≥ 16) is used as an indicator of depression, only Generation Zs are included, and personal factors such as personhood are used in addition to occupational stress. The use of personal factors, such as personality, in addition to occupational stress, was considered the reason for the high accuracy, as it differed significantly from the LTSA-MD in this study. In contrast, in a study using the BJSQ of 164 university staff on long-term psychiatric sick leave and 307 subjects matched on gender, age, and job category, a logistic regression analysis was used to create a predictive model. Factors were identified, and the AUC of the ROC curve was 0.768, indicating that the performance of the prediction model was modest^26^. In another study using BJSQ data and health examination results data from a Japanese manufacturing company (approximately 3000 employees), a random forest model was used to determine whether having a high stress response score on the BJSQ, being young, and belonging to a specific department were significant predictors of illness due to mental illness as important predictors of leave (ROC curve AUC = 0.882), and an AUC of 0.607 for physical illness^20^. These two studies used methods similar to those used in this study, and both used the ROC curve AUC as their assessment method. Their values showed similar or higher predictive power than the ROC curve (AUC) in this study.

However, LTSA-MD is a very low-probability event (0.53%), and is difficult to learn and predict. In such imbalanced data, predicting all of them as non-LTSA-MD results in very high specificity and accuracy, which makes the predictive ability appear high. In the above study, this problem was avoided by undersampling, and the predictive ability was evaluated using the AUC of the ROC curve, which may have resulted in a relatively high value. However, as an actual adaptation to the field, imbalanced data are used to make predictions. Therefore, the data used to make predictions cannot be undersampled. In this study, multiple sampling methods were used during training, imbalanced data were used as prediction data, and the AP was calculated in addition to the AUC of the ROC to focus on the forecasting performance of a small number of classes as a method for evaluating predictive ability. The PR curve indicates how accurately a model can predict a small number of classes, whereas the performance of a large number of classes is not considered. Although most machine learning and sampling methods gave a very low AP results, the gradient boosted trees model and bootstrap oversampling method demonstrated highest AP among all integrations of machine learning and sampling methods, with a AP of 0.040 and a ROC-AUC of 0.81.

Furthermore, when important features for this combination were calculated using SHAP, the top features were dominated by background factors (occupation, age, manager, gender composition, number of organizational members), while overall, items related to stress reactions were prevalent. This aligns with the notion that improving environmental factors contributes to preventing LTSA-MD and also corresponds with the fact that stress reactions serve as the most powerful screening criterion for identifying high-stress individuals within Japan’s stress check system.

### Strengths and contributions

This study has several notable strengths compared with previous work. First, we analyzed a large-scale dataset of more than 230,000 Japanese public servants, which enabled us to investigate the rare outcome of LTSA-MD. Second, we systematically compared five machine learning algorithms combined with six different resampling methods, providing one of the most comprehensive evaluations of strategies to address severe class imbalance. Third, unlike most previous studies that used selected stress indicators, we utilized all BJSQ items to capture the multidimensional aspects of occupational stress. Finally, in addition to conventional ROC AUC, we evaluated PRC AUC and sensitivity, which are particularly informative for rare-event prediction.

### Limitations

Our study, while providing valuable insights, has also certain limitations. First, the reliance on self-reported data may have introduced subjective bias. Second, the specific context of Japanese public servants may limit the generalizability of our findings to other occupational groups or cultural settings. Third, there is a duration of one day to a maximum of one year between the BJSQ response date and the LTSA-MD start date. This difference in duration may have affected the results. Fouth, although we tested supplementary predictors from health checkup data, including blood test results and physiological measurements, as well as ensemble approaches such as stacking and voting classifiers. However, these attempts did not substantially improve predictive performance, suggesting that the rarity of LTSA-MD remains a fundamental challenge for prediction. Fifth, our study used only existing data and could not obtain data about the severity, duration, or recurrence of participants. Future studies should address these clinically important aspects to provide a more comprehensive understanding of LTSA-MD. Sixth, the dataset consisted exclusively of Japanese public servants. Occupational cultures, health policies, and stressor profiles may differ substantially across industries and countries. Therefore, external validation using data from diverse occupational groups and international cohorts will be essential for confirming the robustness of our predictive models.

### Implications and future directions

This study explores the use of various machine learning methods to predict LTSA-MD using job stressor data, and the health examination results of workers in Japan. Despite employing a range of machine-learning models and sampling techniques, the overall predictive performance was low, particularly for the minority class of LTSA-MD. The gradient boosted trees model and bootstrap oversampling method yielded a AP of 0.040 and a ROC-AUC of 0.81; however, this was still modest. These results suggest that while job stressor data may be linked to LTSA-MD, the current methods and datasets may not be sufficient for accurate predictions. Future research should explore additional variables, such as personal factors, psychosocial factors, and health check data, adaptation of ensemble learning by combining multiple machine learning methods, and address the limitations related to self-reported data and the timing of stressor assessments relative to the onset of LTSA-MD. Improving these aspects may enhance the utility of machine learning models for predicting mental-health-related absenteeism in the workplace.

We will focus on practical external checks. First, we will test the same model in a later year within the same organization to see if performance is stable over time. Second, we will test it in other workplaces in Japan (public and private) using the same variables and code. Third, we will explore one international partner to verify portability. If performance drops, we will refit the model on the new data and recheck one operating threshold. Where available, we will also add routinely collected information (e.g., health check items) to see if this improves performance in a simple, reproducible way.

## Methods

### Study design

A retrospective observational design was selected to comprehensively analyze existing data, allowing for a large sample size and broad generalizability. We enrolled public servants aged 18–65, working at either the municipal or ward office of City A, situated in the Kinki region of Japan, from 2011 to 2022.

### Participants

Participants included Japanese public servants who responded to the Brief Job Stress Questionnaire (BJSQ) from 2011 to 2014 as part of the original mental health promotion program, or since 2015 as part of the Stress Check Program each year, in which the Japanese government instituted an occupational health policy to identify individuals experiencing high psychosocial stress in workplaces with 50 or more employees. We received secondary data from participants who responded to the BJSQ in total from 2011 to 2022 with an encrypted ID (*n* = 236,172), which had been previously anonymized by office staff in City A. We excluded participants who were not between the ages of 20 and 65 (*n* = 4,747), and questionnaires with incomplete responses or information (*n* = 10,580). Consequently, data from 220,845 workers (94.0%) from 2011 to 2022 were analyzed to determine their eligibility for inclusion in the study. Table 1 shows the number of participants and the intervals of BJSQ responses for each year.

### Outcome variable

The outcome variable was the number of participants who underwent an LTSA between 2011 and 2022. The LTSA data were obtained anonymously from employment records with an encrypted ID. Employers in City A were required to provide a medical certificate issued by a doctor when taking a leave of absence of 90 days or more. The medical certificate for each LTSA was converted to the ICD-10 code by researchers with more than 10 years of clinical experience. LTSA-MD was defined as any sickness absence exceeding 90 days, coded under F00-F99 according to the International Classification of Diseases, Tenth Revision (ICD-10) criteria^21^. Table 2 shows the ICD-10 codes of the LTSA-MD workers.

### Predictor variables

The predictor variables comprised a set of sociodemographic data, BJSQ answers.

### Sociodemographic data

Sociodemographic variables collected included gender, age, and number of organizational members as demographic variables, and job rank (non-manager and manager) and job categories (clerical, technical, professional, and contract workers). Clerical workers were defined as individuals engaged in clerical tasks related to the construction, design, and management (among various roles) of buildings in the municipality; technical workers were those involved in technical tasks requiring physical efforts in the municipality; professional workers included nurses, care workers, public health nurses, and childcare workers.

### Brief Job Stress Questionnaire

The BJSQ subscales were used for ML analysis. The BJSQ was initially derived from the Job Content Questionnaire^27^ and the Generic Job Stress Questionnaire^28^ developed by the National Institute for US Occupational Safety and Health. An extensive inquiry involving Japanese workers substantiated the questionnaire’s validity and reliability^29^. Comprising 57 items, the BJSQ utilized a four-point Likert scale ranging from 1 (disagree) to 4 (agree). These items were systematically categorized into the following dimensions: job stressors (17 items; score range: 17–68), stress responses (29 items; score range: 29–116), social support (nine items; score range: 9–36), and work and life satisfaction (two items; score range: 2–8). Furthermore, these dimensions have their own subscales. Job stressors included psychological stressors pertinent to work, including quantitative workload (three items), qualitative workload (three items), physical demands (one item), interpersonal conflict (three items), poor physical environment (one item), job control (three items), skill utilization (one item), suitable jobs (one item), and meaningfulness of work (one item). Stress responses delineated psychological and physiological reactions to stress, including vigor (3 items), irritability (3 items), fatigue (3 items), anxiety (3 items), depression (6 items), and physical stress response (11 items). Social support measures supportive elements within the workplace, comprising support from supervisors (three items), coworkers (three items), and family or friends (three items). Job satisfaction comprised satisfaction with work and family life (two items). In the computation of BJSQ scores, reversed scoring was selectively applied as needed, with higher scores on each BJSQ subscale indicating elevated stress levels. The Cronbach’s alpha coefficients for the factors and subscales were as follows: 0.80 for occupational stressors, 0.94 for stress response, 0.89 for social support, and 0.53 for work and life satisfaction. We used the 57 items of the BJSQ before calculating them as continuous variables in the machine learning models.

### Statistical analysis

#### Machine learning models

Five different ML models were considered to predict LTSA-MD incidents: logistic regression, random forest, support vector machine (SVM), multilayer perceptron (MLP), and gradient boosted trees (GBT). Random forest and gradient boosting models were selected for their robustness to overfitting and ability to handle complex, nonlinear relationships in the data. In each model, 70% of the total sample was stratified by LTSA-MD rates and selected as training data, while the remaining 30% was used as test data to predict LTSA-MD.

Logistic regression is a widely used statistical prediction model for binary classifications. Logistic regression predicts the chance of classification into one of two groups when the response variable is binary, based on a set of covariate values. The model is advantageous in that the calculated coefficients represent log odds ratios.

Random forest is a quintessential classification technique in ensemble models, comprising numerous decision trees. The ensemble method amalgamates the prediction outcomes from multiple algorithms to enhance predictive accuracy while mitigating overfitting. Random forest consolidates the predictive outcomes from numerous decision trees to arrive at a final determination.

Support Vector Machine (SVM) is a machine learning method used for binary classification tasks. The SVM aims to establish a decision boundary that effectively distinguishes between two categories of data. SVM is recognized for its efficacy in high-dimensional data scenarios, but its computing expense remains low compared with other machine learning techniques^30^.

A Multilayer Perceptron (MLP) is a form of an artificial neural network with a minimum of three layers: an input layer, one or more hidden layers, and an output layer. Each layer comprises interlinked nodes or neurons. MLPs employ nonlinear activation functions such as sigmoid or ReLU to facilitate the network’s ability to learn intricate patterns in the input. They are trained using a technique known as backpropagation, which modifies the weights of the connections to reduce the discrepancies between the expected and actual outputs. MLPs are extensively employed for classification, regression, and pattern recognition across several domains, including image and speech recognition^31^.

GBT is a powerful machine learning technique used for both classification and regression tasks. It combines the predictions of multiple decision trees to improve the accuracy and performance of the model. The core idea behind GBT is to build models sequentially, with each new model correcting the errors of the previous models. This is achieved through a process called boosting, where each tree is trained on the residuals of previous trees, effectively minimizing the overall prediction error^32^.

### Sampling methods

The original dataset was highly imbalanced, with LTSA-MD cases representing only 0.5% of the total sample (1,216 cases among 231,425 participants). This imbalance presents a major challenge for predictive modeling, as models may default to predicting the majority (non-LTSA-MD) class, resulting in poor sensitivity. To address this imbalance, we employed four different sampling strategies: (i) Random sampling, in which 10% of the non-LTSA-MD cases were randomly selected to reduce class imbalance; (ii) Equal size sampling, where the LTSA-MD and non-LTSA-MD groups were matched to have the same sample size; (iii) Synthetic Minority Oversampling Technique (SMOTE), which generates synthetic samples of the minority class; (iv) Bootstrapping, in which minority class cases were randomly duplicated to balance the dataset; (v) Borderline-SMOTE, which focuses on generating synthetic samples near the decision boundary between classes; and (vi) Adaptive Synthetic Sampling (ADASYN), which adaptively generates more synthetic samples for minority instances that are difficult to classify, based on their local distribution. By systematically applying and comparing these six methods, we aimed to evaluate how different resampling strategies influence model performance in the context of highly imbalanced LTSA-MD prediction.

Various measures, including accuracy, precision, sensitivity, specificity, F-value, Average Precision (AP), and the Receiver Operating Characteristic (ROC) curve with Area Under Curve (AUC), were calculated to compare the prediction model performance. The PRC and AUC metrics were chosen owing to their effectiveness in evaluating predictive performance in imbalanced datasets, such as those used in this study. We computed 95% confidence intervals for AP and ROC-AUC via class-stratified bootstrap (B = 200; percentile method), using the same train/test split and seeds as the primary analysis. To identify the most promising model–sampling combinations under severe class imbalance, we prioritized AP and selected the top 10 combinations by AP as candidates for statistical comparison. For classifier comparisons, predictions were binarized by top-k (k equal to the number of positives in the test set, k = 350). Pairwise McNemar tests were planned only if Cochran’s Q indicated significant heterogeneity (α = 0.05), evaluated on identical cases (overall and, as a sensitivity analysis, positives-only).

To render model predictions interpretable and verify face validity, we used Shapley additive explanation (SHAP) to identify which BJSQ items and background covariates most contributed to LTSA-MD risk^33^. SHAP assigns each feature a per-instance contribution (Shapley value) such that the sum of contributions equals the model’s prediction relative to a baseline (additivity/local accuracy); positive values raise, and negative values lower, the predicted LTSA-MD risk. We computed SHAP values to interpret the top-ranked model. Mean absolute SHAP values were calculated on the common test set and aggregated per feature; contributions were also grouped into BJSQ domains (job stressors, stress responses, social supports, and satisfaction) and background variables.

All statistical analyses were performed using KNIME version 5.3.0 (KNIME AG, Zurich, Switzerland) and Python (Python Software Foundation, Version 3.12.4).

## Conclusion

This study assesses the predictive ability of various machine learning models to identify LTSA-MD using job stressor data from Japanese workers. Despite testing multiple models and sampling methods, the predictive performance was generally low, with logistic regression and SMOTE oversampling showing relatively higher, but modest accuracy. These findings suggest that job stressor data alone may not be sufficient to accurately predict LTSA-MD. Future work should focus on incorporating additional factors, such as health checkup data, to improve prediction accuracy.

### Ethical considerations

The Human Subjects Review Committee approved the study protocol (authorization number: 3337). As the study was conducted retrospectively using pre-existing, anonymized data, it did not involve direct participation from individuals, and due to retrospective nature of the study The informed consent was waived by the Ethical Committee of Osaka Metropolitan University Graduate School of Medicine review board. To resolve possible participants’ anxiety regarding the use of sensitive personal documents, information about this study was built into our department’s website, and the opportunity to opt out of the study was provided to potential participants. In addition to anonymization, data was stored on secure servers, and access was restricted to authorized personnel only, ensuring compliance with national data protection regulations. All methods were carried out in accordance with relevant guidelines and regulations.

## Supplementary Information

Below is the link to the electronic supplementary material.

Supplementary Material 1

Supplementary Material 2

## Data Availability

The data presented in this study are available in this article. Additional data that support the findings of this study are available upon reasonable request from the corresponding author, with permission from the data provider. Owing to confidentiality agreements, the raw data underlying the results are not publicly available.

## Code availability

The KNIME workflows used to preprocess the data, train the machine learning models, compute performance metrics (including bootstrapped confidence intervals), and generate the tables and figures are provided as Supplementary Code S1. The workflows include all embedded Python scripts used in the analyses.
