# Supplementary material for: Machine learning prediction of long-term sickness absence due to mental disorders using Brief Job Stress Questionnaire data
Source: Sci Rep. 2025 Dec 16;16:2908. doi: 10.1038/s41598-025-32857-3 (PMC12830388; doi:10.1038/s41598-025-32857-3)
Supplement: Supplementary file 1 — Supplementary Material 1 [file 41598_2025_32857_MOESM1_ESM.pdf]

Supplementary Table S1. Complete grid of algorithm×sampling results.

Algorithm	Sampling Method	Precision	Sensitivity	Specificity	F1	Accuracy	MCC	ROC(AUC)	ROC(95%CI)	PRC-AP	PRC-AP(95%CI)
Logistic Regression model	No Sampling	0.01	0.01	1.00	0.01	0.99	0.00	0.44	(0.40 - 0.47)	0.005	(0.004 - 0.006)
	Random Sampling	0.03	0.29	0.95	0.05	0.95	0.08	0.75	(0.73 - 0.78)	0.022	(0.017 - 0.028)
	SMOTE	0.03	0.25	0.95	0.05	0.94	0.06	0.74	(0.71 - 0.76)	0.018	(0.014 - 0.022)
	Equal Size Sampling	0.02	0.40	0.91	0.04	0.91	0.08	0.76	(0.74 - 0.79)	0.020	(0.016 - 0.025)
	Bootstrap Sampling X10	-	0.00	1.00	-	0.99	-	0.67	(0.64 - 0.69)	0.010	(0.008 - 0.013)
	Borderline-SMOTE	0.01	0.07	0.96	0.02	0.96	0.01	0.60	(0.57 - 0.63)	0.008	(0.007 - 0.011)
	ADASYN	-	0.00	1.00	-	0.99	-	0.61	(0.58 - 0.64)	0.008	(0.007 - 0.010)
Random Forest	No Sampling	-	0.00	1.00	-	0.99	-	0.60	(0.57 - 0.62)	0.020	(0.011 - 0.033)
	Random Sampling	0.00	0.00	1.00	-	0.99	0.00	0.74	(0.71 - 0.77)	0.033	(0.023 - 0.048)
	SMOTE	0.04	0.17	0.98	0.07	0.97	0.07	0.78	(0.75 - 0.80)	0.027	(0.021 - 0.037)
	Equal Size Sampling	0.02	0.67	0.78	0.03	0.78	0.08	0.79	(0.77 - 0.81)	0.025	(0.020 - 0.032)
	Bootstrap Sampling X10	1.00	0.00	1.00	0.01	0.99	0.05	0.71	(0.68 - 0.74)	0.036	(0.024 - 0.054)
	Borderline-SMOTE	0.04	0.12	0.99	0.06	0.98	0.06	0.76	(0.73 - 0.79)	0.025	(0.020 - 0.033)
	ADASYN	0.04	0.23	0.97	0.06	0.97	0.08	0.77	(0.75 - 0.80)	0.026	(0.020 - 0.036)
Support Vector Machine	No Sampling	1.00	0.03	1.00	0.06	0.99	0.17	0.50	(0.49 - 0.52)	0.038	(0.020 - 0.058)
	Random Sampling	0.39	0.04	1.00	0.07	0.99	0.12	0.51	(0.50 - 0.53)	0.021	(0.011 - 0.039)
	SMOTE	0.89	0.02	1.00	0.04	0.99	0.14	0.49	(0.48 - 0.51)	0.028	(0.014 - 0.047)
	Equal Size Sampling	0.16	0.04	1.00	0.06	0.99	0.08	0.52	(0.51 - 0.53)	0.027	(0.013 - 0.046)
	Bootstrap Sampling X10	-	0.00	1.00	-	0.99	-	0.47	(0.45 - 0.48)	0.005	(0.004 - 0.005)
	Borderline-SMOTE	0.26	0.04	1.00	0.07	0.99	0.10	0.51	(0.50 - 0.53)	0.016	(0.009 - 0.029)
	ADASYN	0.21	0.04	1.00	0.07	0.99	0.09	0.51	(0.50 - 0.53)	0.018	(0.010 - 0.034)
Multi Layer Perception	No Sampling	-	0.00	1.00	-	0.99	-	0.74	(0.72 - 0.77)	0.027	(0.021 - 0.037)
	Random Sampling	0.07	0.03	1.00	0.04	0.99	0.04	0.67	(0.65 - 0.70)	0.014	(0.010 - 0.022)
	SMOTE	0.02	0.59	0.81	0.03	0.81	0.07	0.76	(0.74 - 0.79)	0.022	(0.017 - 0.027)
	Equal Size Sampling	0.01	0.72	0.68	0.02	0.68	0.06	0.76	(0.74 - 0.79)	0.021	(0.017 - 0.029)
	Bootstrap Sampling X10	0.00	0.00	1.00	-	0.99	0.00	0.76	(0.73 - 0.79)	0.027	(0.021 - 0.037)
	Borderline-SMOTE	0.02	0.55	0.84	0.04	0.84	0.08	0.75	(0.72 - 0.78)	0.026	(0.021 - 0.035)
	ADASYN	0.01	0.73	0.66	0.02	0.66	0.06	0.77	(0.75 - 0.80)	0.030	(0.022 - 0.042)
Gradient Boosted Trees	No Sampling	0.00	0.00	1.00	-	0.99	0.00	0.80	(0.78 - 0.83)	0.032	(0.025 - 0.043)
	Random Sampling	0.06	0.03	1.00	0.04	0.99	0.04	0.80	(0.78 - 0.83)	0.035	(0.027 - 0.049)
	SMOTE	0.05	0.03	1.00	0.04	0.99	0.04	0.76	(0.74 - 0.79)	0.026	(0.020 - 0.037)

Equal Size Sampling	0.01	0.72	0.72	0.03	0.72	0.07	0.79	(0.77 - 0.81)	0.024	(0.020 - 0.034)
Bootstrap Sampling X10	0.14	0.03	1.00	0.05	0.99	0.07	0.81	(0.78 - 0.83)	0.040	(0.029 - 0.058)
Borderline-SMOTE	0.06	0.05	1.00	0.05	0.99	0.05	0.73	(0.70 - 0.76)	0.026	(0.020 - 0.036)
ADASYN	0.04	0.03	1.00	0.03	0.99	0.03	0.76	(0.73 - 0.78)	0.025	(0.019 - 0.036)

BJSQ: Brief Job Stress Questionnaire; SMOTE: Synthetic Minority Over-sampling Technique; ADASYN: Adaptive Synthetic Sampling.

Hyphen: All data were predicted to be non-long-term illness absences due to mental disorders; therefore, they were not calculated.

Supplementary Table S2. Average precision (AP) with 95% bootstrapped CIs (percentile; B=200) and ROC-AUC for the AP top-10 model–sampling combinations on the common test set.

Rank	Sampling Method	Algorithm	AP	95% CI	ROC(AUC)
1	Bootstrap Sampling X10	Gradient Boosted Trees	0.040	(0.029 - 0.058)	0.81
2	No Sampling	Support Vector Machine	0.038	(0.020 - 0.058)	0.50
3	Bootstrap Sampling X10	Random Forest	0.036	(0.024 - 0.054)	0.71
4	Random Sampling	Gradient Boosted Trees	0.035	(0.027 - 0.049)	0.80
5	Random Sampling	Random Forest	0.033	(0.023 - 0.048)	0.74
6	No Sampling	Gradient Boosted Trees	0.032	(0.025 - 0.043)	0.80
7	ADASYN	Multi Layer Perception	0.030	(0.022 - 0.042)	0.77
8	SMOTE	Support Vector Machine	0.028	(0.014 - 0.047)	0.49
9	SMOTE	Random Forest	0.027	(0.021 - 0.037)	0.78
10	Bootstrap Sampling X10	Multi Layer Perception	0.027	(0.021 - 0.037)	0.76

Supplementary Table S3. the performance of machine learning algorithms and sampling methods for predicting long-term sickness absences due to mental disorders by BJSQ subscale scores.

Algorithm	Sampling Method	Precision	Sensitivity	Specificity	F-measure	Accuracy	MCC	ROC(AUC)	ROC(95%CI)	PRC-AP	PRC-AP(95%CI)
Logistic Regression model	No Sampling	-	0.00	1.00	-	0.99	-	0.58	(0.56 - 0.61)	0.007	(0.006 - 0.011)
	10% Random Sampling	-	0.00	1.00	-	0.99	-	0.62	(0.60 - 0.65)	0.010	(0.007 - 0.018)
	SMOTE	0.01	0.79	0.51	0.02	0.52	0.04	0.69	(0.66 - 0.71)	0.009	(0.008 - 0.010)
	Equal Size Sampling	0.02	0.09	0.98	0.04	0.98	0.04	0.73	(0.70 - 0.75)	0.015	(0.012 - 0.020)
	Bootstrap Sampling X10	-	0.00	1.00	-	0.99	-	0.62	(0.60 - 0.65)	0.009	(0.007 - 0.015)
	Borderline-SMOTE	0.02	0.47	0.84	0.03	0.84	0.06	0.72	(0.69 - 0.74)	0.013	(0.011 - 0.016)
	ADASYN	0.01	0.30	0.89	0.03	0.89	0.05	0.69	(0.66 - 0.72)	0.014	(0.011 - 0.017)
Random Forest	No Sampling	-	0.00	1.00	-	0.99	-	0.59	(0.57 - 0.61)	0.020	(0.012 - 0.033)
	10% Random Sampling	0.00	0.00	1.00	-	0.99	0.00	0.72	(0.69 - 0.75)	0.029	(0.020 - 0.045)
	SMOTE	0.03	0.33	0.94	0.05	0.94	0.09	0.77	(0.74 - 0.80)	0.026	(0.020 - 0.036)
	Equal Size Sampling	0.01	0.67	0.76	0.03	0.76	0.07	0.78	(0.76 - 0.81)	0.026	(0.021 - 0.035)
	Bootstrap Sampling X10	1.00	0.00	1.00	0.01	0.99	0.05	0.69	(0.66 - 0.72)	0.036	(0.024 - 0.054)
	Borderline-SMOTE	0.04	0.12	0.98	0.06	0.98	0.06	0.74	(0.71 - 0.77)	0.022	(0.017 - 0.031)
	ADASYN	0.03	0.18	0.97	0.05	0.97	0.06	0.76	(0.73 - 0.78)	0.022	(0.016 - 0.031)
Support Vector Machine	No Sampling	1.00	0.03	1.00	0.06	0.99	0.17	0.62	(0.59 - 0.65)	0.043	(0.024 - 0.064)
	10% Random Sampling	0.39	0.04	1.00	0.99	0.99	0.12	0.62	(0.59 - 0.65)	0.022	(0.013 - 0.039)
	SMOTE	0.91	0.03	1.00	0.06	0.99	0.16	0.60	(0.57 - 0.63)	0.037	(0.021 - 0.057)
	Equal Size Sampling	0.15	0.04	1.00	0.06	0.99	0.08	0.56	(0.53 - 0.58)	0.026	(0.013 - 0.046)
	Bootstrap Sampling X10	-	0.00	1.00	-	0.99	-	0.57	(0.54 - 0.60)	0.007	(0.006 - 0.009)
	Borderline-SMOTE	0.31	0.04	1.00	0.07	0.99	0.11	0.63	(0.59 - 0.65)	0.021	(0.013 - 0.036)
	ADASYN	0.29	0.04	1.00	0.07	0.99	0.11	0.62	(0.59 - 0.65)	0.023	(0.014 - 0.041)
Multi Layer Perception	No Sampling	-	0.00	1.00	-	0.99	-	0.75	(0.72 - 0.77)	0.023	(0.017 - 0.029)
	10% Random Sampling	0.06	0.02	1.00	0.03	0.99	0.04	0.74	(0.71 - 0.77)	0.023	(0.017 - 0.032)
	SMOTE	0.01	0.67	0.71	0.02	0.71	0.06	0.75	(0.73 - 0.78)	0.024	(0.017 - 0.037)
	Equal Size Sampling	0.01	0.69	0.70	0.02	0.70	0.06	0.76	(0.73 - 0.78)	0.020	(0.016 - 0.025)
	Bootstrap Sampling X10	0.12	0.02	1.00	0.03	0.99	0.04	0.77	(0.74 - 0.79)	0.025	(0.019 - 0.034)
	Borderline-SMOTE	0.02	0.49	0.83	0.03	0.83	0.06	0.73	(0.71 - 0.76)	0.021	(0.017 - 0.031)
	ADASYN	0.01	0.73	0.70	0.02	0.70	0.07	0.77	(0.74 - 0.79)	0.022	(0.018 - 0.031)
Gradient Boosted Leaner	No Sampling	0.11	0.00	1.00	0.01	0.99	0.02	0.79	(0.77 - 0.81)	0.033	(0.025 - 0.047)
	10% Random Sampling	0.04	0.02	1.00	0.02	0.99	0.02	0.79	(0.77 - 0.81)	0.027	(0.022 - 0.035)
	SMOTE	0.04	0.23	0.97	0.07	0.97	0.09	0.78	(0.75 - 0.80)	0.028	(0.023 - 0.037)
	Equal Size Sampling	0.01	0.70	0.71	0.02	0.71	0.06	0.77	(0.75 - 0.80)	0.023	(0.018 - 0.029)
	Bootstrap Sampling X10	0.13	0.02	1.00	0.03	0.99	0.05	0.79	(0.76 - 0.81)	0.033	(0.025 - 0.047)

	Borderline-SMOTE	0.06	0.07	0.99	0.07	0.99	0.06	0.73	(0.70 - 0.75)	0.023	(0.017 - 0.034)
	ADASYN	0.05	0.03	1.00	0.04	0.99	0.03	0.74	(0.71 - 0.76)	0.023	(0.018 - 0.033)

BJSQ: Brief Job Stress Questionnaire, SMOTE: Synthetic Minority Oversampling Technique.

Hyphen: All data were predicted to be non-long-term illness absences due to mental disorders; therefore, they were not calculated.

Supplementary Figure S1. Average precision heatmap – all algorithm × sampling combinations

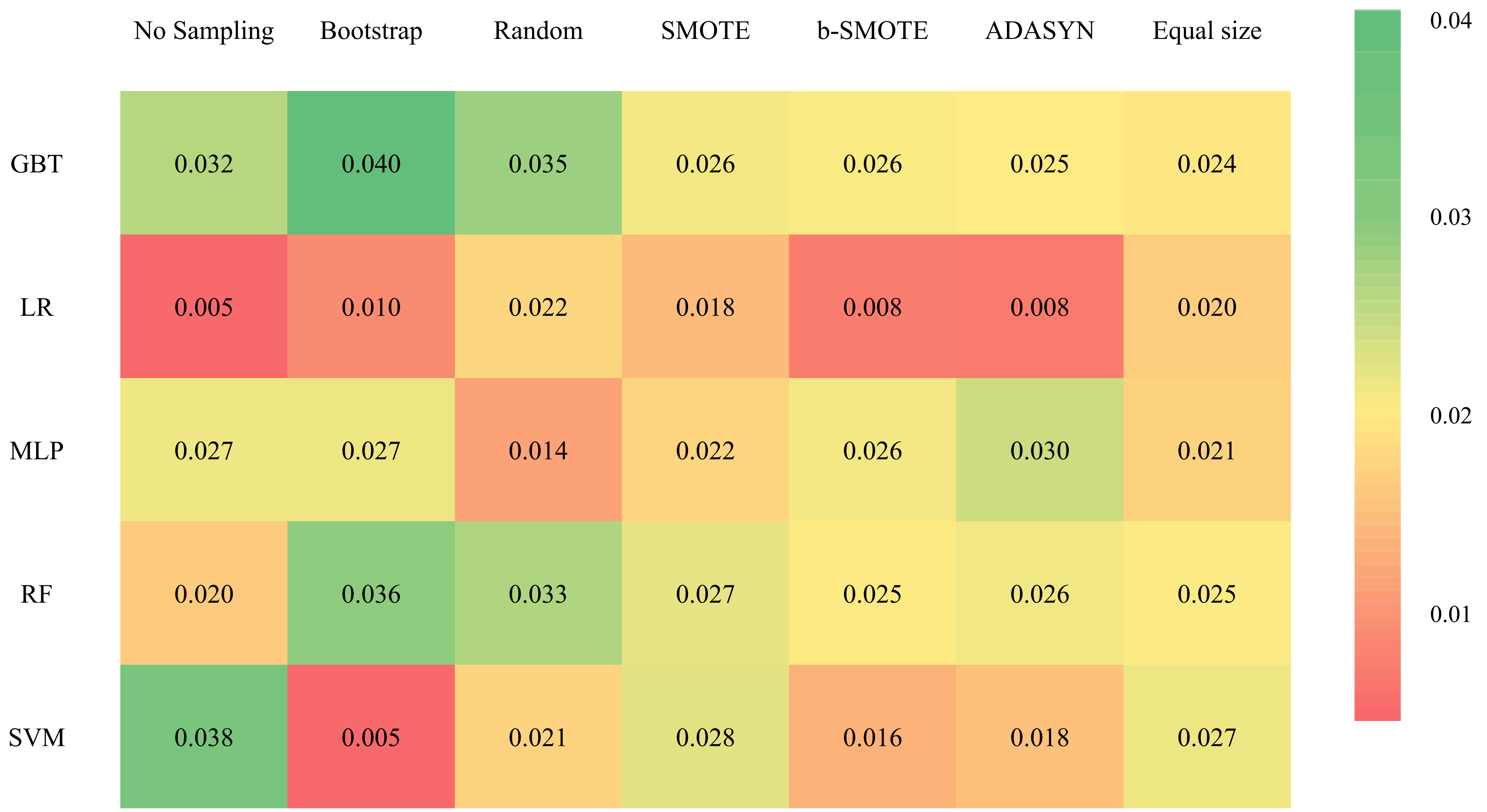

Cells display average precision; higher is better.

Abbreviations — Algorithms: LR: Logistic Regression; SVM: Support Vector Machine; RF: Random Forest; GBT: Gradient Boosted Trees; MLP: Multilayer Perceptron. Sampling: Bootstrap: Bootstrap 10 resamples; Random: 10% random sampling, SMOTE: Synthetic Minority Over-sampling Technique; b-SMOTE: Borderline-SMOTE; ADASYN: Adaptive Synthetic sampling; Equal size: class-balanced sampling.
