# Supplementary material for: Machine learning prediction of long-term sickness absence due to mental disorders using Brief Job Stress Questionnaire data
Source: Sci Rep. 2025 Dec 16;16:2908. doi: 10.1038/s41598-025-32857-3 (PMC12830388; doi:10.1038/s41598-025-32857-3)
Supplement: Supplementary file 2 — Supplementary Material 2 [file 41598_2025_32857_MOESM2_ESM.zip › Codes/Precision-Recall.py]

# --- KNIME Python Script: AP（verage Precision） PR AUC（台形積分） ROC AUC + 95%CI + 曲線点（ロング表）---# 入力  Port0 = 推論結果テーブル（列 MentalSL, P (MentalSL=1)） 出力  Port0 = "metrics / bootstrap / pr_curve / roc_curve" のロング表 依存 scikit-learnimport numpy as npimport pandas as pdfrom sklearn.metrics import average_precision_score, precision_recall_curve, aucfrom sklearn.metrics import roc_auc_score, roc_curveimport knime.scripting.io as knio# ====== 設定======TARGET_COL = "SL"           # 正解ラベル（/1）ROBA_COL  = "P (SL=1)"     # 陽性クラスの確率列          = 1000                 # ブートストラップ反復（配線確認時は小さめに）EED       = 42                   # 乱数シード ===================# --- 入力取得---df = knio.input_tables[0].to_pandas()# 必須列チェックissing = [c for c in [TARGET_COL, PROBA_COL] if c not in df.columns]if missing:    out = pd.DataFrame({"panel": ["error"], "message": [f"必要列が見つかりません {missing}"]})    knio.output_tables[0] = knio.Table.from_pandas(out)else:    # 型整備   y_true  = pd.to_numeric(df[TARGET_COL], errors="coerce").astype("Int64").astype("float").to_numpy()    y_score = pd.to_numeric(df[PROBA_COL],  errors="coerce").astype(float).to_numpy()    # 欠損除去   mask = np.isfinite(y_true) & np.isfinite(y_score)    y_true = y_true[mask].astype(int)    y_score = y_score[mask].astype(float)    n = len(y_true)    B_eff = int(B) if n > 0 else 0    classes = np.unique(y_true)    # --- 点推定（P とPR AUC台形、OC AUC）--    # PR曲線（点推定用）   prec, rec, _ = precision_recall_curve(y_true, y_score)  # x=recall(昇順, y=precision    ap_base = average_precision_score(y_true, y_score)       # AP（推奨）   pr_auc_trap_base = auc(rec, prec)                        # 台形積分のR AUC    try:        roc_base = roc_auc_score(y_true, y_score) if len(classes) >= 2 else np.nan    except Exception:        roc_base = np.nan    # --- ブートストラップ---    rng = np.random.RandomState(SEED)    aps   = np.full(B_eff, np.nan, dtype=float)    prts  = np.full(B_eff, np.nan, dtype=float)  # PR trapezoid    rocs  = np.full(B_eff, np.nan, dtype=float)    for i in range(B_eff):        idx = rng.choice(n, size=n, replace=True)        yt, ys = y_true[idx], y_score[idx]        # AP        try:            aps[i] = average_precision_score(yt, ys)        except Exception:            aps[i] = np.nan        # PR AUC (台形        try:            p_i, r_i, _ = precision_recall_curve(yt, ys)            prts[i] = auc(r_i, p_i)        except Exception:            prts[i] = np.nan        # ROC AUC        try:            rocs[i] = roc_auc_score(yt, ys)        except Exception:            rocs[i] = np.nan    def ci_summary(arr: np.ndarray):        arr = np.asarray(arr, dtype=float)        arr = arr[~np.isnan(arr)]        if arr.size == 0:            return dict(lo=np.nan, hi=np.nan, med=np.nan, mean=np.nan, n_valid=0)        lo, hi = np.percentile(arr, [2.5, 97.5])        return dict(lo=float(lo), hi=float(hi), med=float(np.median(arr)),                    mean=float(np.mean(arr)), n_valid=int(arr.size))    ap_sum   = ci_summary(aps)    prt_sum  = ci_summary(prts)    roc_sum  = ci_summary(rocs)    # --- ROC曲線点（点推定）--    try:        fpr, tpr, _ = roc_curve(y_true, y_score)    except Exception:        fpr, tpr = np.array([np.nan]), np.array([np.nan])    # --- 出力ロング表---    # パネル：メトリクス集約（点推定＋5%CI）   metrics = pd.DataFrame({        "panel":   ["metrics",            "metrics",               "metrics"],        "metric":  ["PR AUC (AP)",        "PR AUC (trapezoid)",    "ROC AUC"],        "point_estimate": [ap_base,       pr_auc_trap_base,        roc_base],        "ci_lower_95":    [ap_sum["lo"],  prt_sum["lo"],           roc_sum["lo"]],        "ci_upper_95":    [ap_sum["hi"],  prt_sum["hi"],           roc_sum["hi"]],        "median":         [ap_sum["med"], prt_sum["med"],          roc_sum["med"]],        "mean":           [ap_sum["mean"],prt_sum["mean"],         roc_sum["mean"]],        "n_boot_valid":   [ap_sum["n_valid"], prt_sum["n_valid"],  roc_sum["n_valid"]],        "B":              [B_eff,         B_eff,                   B_eff]    })    # パネル：ブートストラップ分布   boot = pd.DataFrame({        "panel":   "bootstrap",        "iter":    np.arange(1, B_eff+1),        "ap":      aps if B_eff > 0 else [],        "pr_auc_trap": prts if B_eff > 0 else [],        "roc_auc": rocs if B_eff > 0 else [],    })    # パネル：R曲線点（点推定）   pr_curve_df = pd.DataFrame({        "panel": "pr_curve",        "x": rec, "y": prec    })    # パネル：OC曲線点（点推定）   roc_curve_df = pd.DataFrame({        "panel": "roc_curve",        "x": fpr, "y": tpr    })    out_long = pd.concat([metrics, boot, pr_curve_df, roc_curve_df], ignore_index=True, sort=False)    knio.output_tables[0] = knio.Table.from_pandas(out_long)
