# Supplementary material for: Machine learning prediction of long-term sickness absence due to mental disorders using Brief Job Stress Questionnaire data
Source: Sci Rep. 2025 Dec 16;16:2908. doi: 10.1038/s41598-025-32857-3 (PMC12830388; doi:10.1038/s41598-025-32857-3)
Supplement: Supplementary file 2 — Supplementary Material 2 [file 41598_2025_32857_MOESM2_ESM.zip › Codes/CochransQ.py]

import knime.scripting.io as knioimport pandas as pdimport numpy as npfrom statsmodels.stats.contingency_tables import cochrans_q# ---- 入力----df = knio.input_tables[0].to_pandas()# ---- 二値列（/1）だけを安全に抽出----num_df = df.select_dtypes(include=["number"]).copy()def is_binary(s: pd.Series) -> bool:    vals = pd.unique(s.dropna())    # 値が{0,1} のみかどうか（loatでもK、例 0.0/1.0）   return set(np.unique(vals)).issubset({0, 1})binary_cols = [c for c in num_df.columns if is_binary(num_df[c])]X = num_df[binary_cols]# Cochran's Q は「同一対象複数条件（列=3）」の/1データが前提f X.shape[1] < 3:    # 列数が足りない場合はエラー詳細を返す（落とさない）   out = pd.DataFrame({        "Q": [np.nan],        "p.value": [np.nan],        "k_conditions": [X.shape[1]],        "n_subjects": [X.shape[0]],        "message": ["Need >=3 binary columns (0/1). Check your input."]    })else:    # ---- Cochran's Q ----    res = cochrans_q(X.to_numpy())  # Bunchオブジェクトが返る   out = pd.DataFrame({        "Q": [res.statistic],        "p.value": [res.pvalue],        "k_conditions": [X.shape[1]],        "n_subjects": [X.shape[0]]    })# ---- 出力----knio.output_tables[0] = knio.Table.from_pandas(out)
