# Supplementary material for: Machine learning prediction of long-term sickness absence due to mental disorders using Brief Job Stress Questionnaire data
Source: Sci Rep. 2025 Dec 16;16:2908. doi: 10.1038/s41598-025-32857-3 (PMC12830388; doi:10.1038/s41598-025-32857-3)
Supplement: Supplementary file 2 — Supplementary Material 2 [file 41598_2025_32857_MOESM2_ESM.zip › Codes/SHAP.py]

import knime.scripting.io as knioimport pandas as pd, numpy as np, re, unicodedatafrom sklearn.compose import ColumnTransformerfrom sklearn.pipeline import Pipelinefrom sklearn.preprocessing import OneHotEncoderfrom sklearn.impute import SimpleImputerfrom xgboost import XGBClassifierfrom sklearn.metrics import average_precision_score, roc_auc_scoreimport xgboost as xgb  # pred_contribs 用 -------------------------------# 1) 強力クリーニング（角括弧E指数全角・配列桁区切り対応） -------------------------------_FLOAT_PAT = re.compile(r'[-+]?(?:\d+(?:\.\d*)?|\.\d+)(?:[eEdD][-+]?\d+)?')def _extract_first_float(x):    if pd.isna(x):        return np.nan    if isinstance(x, (int, float, np.number)):        return float(x)    s = unicodedata.normalize('NFKC', str(x)).strip()    s = s.replace(',', '')  # 桁区切り   # 角丸カッコで囲まれていたら外す   if len(s) >= 2 and (s[0] in '[（' and s[-1] in '])）'):        s = s[1:-1]    # セミコロンカンマスペースで分割→先頭トークンから探索   for token in re.split(r'[;,\|\s]+', s):        m = _FLOAT_PAT.search(token)        if m:            val = m.group(0).replace('D', 'E').replace('d', 'e')            try:                return float(val)            except Exception:                pass    # 埋もれた数値を最後に拾う   m = _FLOAT_PAT.search(s)    if m:        val = m.group(0).replace('D', 'E').replace('d', 'e')        return float(val)    return np.nandef coerce_numeric_like(df: pd.DataFrame, thresh=0.2, verbose=True):    obj_cols = df.select_dtypes(include=['object']).columns.tolist()    converted = []    for c in obj_cols:        s = df[c].astype(str)        # 数値トークンが一定割合以上の列のみ変換       rate = s.str.contains(r'\d', regex=True, na=False).mean()        if rate >= thresh:            df[c] = df[c].map(_extract_first_float)            converted.append((c, round(float(rate), 2)))    if verbose:        print("Converted cols (≥.2f):" % thresh, converted)    return df# -------------------------------# 2) データ読み込み& クリーニング -------------------------------train = knio.input_tables[0].to_pandas()test  = knio.input_tables[1].to_pandas()# 強力クリーニングrain = coerce_numeric_like(train.copy(), thresh=0.2, verbose=True)test  = coerce_numeric_like(test.copy(),  thresh=0.2, verbose=True)# ラベル厳密化rain["SL"] = pd.to_numeric(train["SL"], errors="raise").astype(int)test["SL"]  = pd.to_numeric(test["SL"],  errors="raise").astype(int)y_train = train["SL"].valuesy_test  = test["SL"].valuesX_train = train.drop(columns=["SL"])X_test  = test.drop(columns=["SL"])# 列型の再判定（クリーニング後！）um_cols = X_train.select_dtypes(include=[np.number]).columns.tolist()cat_cols = [c for c in X_train.columns if c not in num_cols]print(f"#num={len(num_cols)}, #cat={len(cat_cols)}")# -------------------------------# 3) ブートストラップ（陽性10） -------------------------------pos_idx = np.where(y_train == 1)[0]rng = np.random.default_rng(42)pos_boot = rng.choice(pos_idx, size=len(pos_idx) * 9, replace=True)  # 既存ぶん9倍計0倍oot_idx = np.concatenate([np.arange(len(y_train)), pos_boot])Xb = X_train.iloc[boot_idx].reset_index(drop=True)yb = y_train[boot_idx]# -------------------------------# 4) 前処理+ 学習（ipeline）    数値 欠損補完（edian）    カテゴリ OneHot（parse=False） -------------------------------pre = ColumnTransformer(    transformers=[        ("num", SimpleImputer(strategy="median"), num_cols),        ("cat", OneHotEncoder(handle_unknown="ignore", sparse=False), cat_cols),    ],    remainder="drop",    sparse_threshold=0.0,)gbm = XGBClassifier(    n_estimators=100, max_depth=5, learning_rate=0.1,    subsample=1.0, colsample_bytree=1.0,    reg_lambda=1.0, objective="binary:logistic",    random_state=42, n_jobs=-1, eval_metric="logloss")pipe = Pipeline([("prep", pre), ("clf", gbm)])pipe.fit(Xb, yb)# 性能確認_test = pipe.predict_proba(X_test)[:, 1]ap = average_precision_score(y_test, p_test)roc = roc_auc_score(y_test, p_test)print(f"AP={ap:.5f}, ROC-AUC={roc:.3f}")# -------------------------------# 5) XGBoost ネイティブSHAP（red_contribs）    →SHAPライブラリのreeExplainerは使わない（ase_score角括弧バグ回避） -------------------------------# 変換後の特徴行列と特徴名test_trans = pipe.named_steps["prep"].transform(X_test)feat_names = list(num_cols)if len(cat_cols):    ohe = pipe.named_steps["prep"].named_transformers_["cat"]    feat_names += ohe.get_feature_names_out(cat_cols).tolist()# Booster から寄与を取得ooster = pipe.named_steps["clf"].get_booster()dtest = xgb.DMatrix(np.asarray(Xtest_trans, dtype=np.float32))contribs = booster.predict(dtest, pred_contribs=True)  # shape: (n_samples, n_features + 1)phi = contribs[:, :-1]  # 最後の列はbias# グローバル重要度（平均SHAP|）mp = pd.DataFrame({    "feature": feat_names,    "mean_abs_shap": np.mean(np.abs(phi), axis=0)}).sort_values("mean_abs_shap", ascending=False)imp.to_csv("shap_global_importance.csv", index=False)print(imp.head(20))# --- KNIME の出力に重要度テーブルを渡す---imp = imp.reset_index(drop=True)knio.output_tables[0] = knio.Table.from_pandas(imp)
