# Supplementary material for: Machine learning prediction of long-term sickness absence due to mental disorders using Brief Job Stress Questionnaire data
Source: Sci Rep. 2025 Dec 16;16:2908. doi: 10.1038/s41598-025-32857-3 (PMC12830388; doi:10.1038/s41598-025-32857-3)
Supplement: Supplementary file 2 — Supplementary Material 2 [file 41598_2025_32857_MOESM2_ESM.zip › Codes/McNemarTest.py]

# KNIME Python Script 用（ython Script(Labs) でも可）mport knime.scripting.io as knioimport pandas as pdimport numpy as npfrom statsmodels.stats.contingency_tables import mcnemar# ========= 1) 入力=========df = knio.input_tables[0].to_pandas()# ========= 2) 0/1 の二値列のみ抽出（D 等の非数値列は除外）========num_df = df.select_dtypes(include=["number"]).copy()def is_binary(s: pd.Series) -> bool:    vals = pd.unique(s.dropna())    if len(vals) == 0:        return False    return set(np.unique(vals)).issubset({0, 1})binary_cols = [c for c in num_df.columns if is_binary(num_df[c])]X = num_df[binary_cols]if X.shape[1] < 2:    out = pd.DataFrame({        "message": ["Need >=2 binary (0/1) columns for McNemar test."],        "k_conditions": [X.shape[1]],        "n_subjects": [X.shape[0]]    })    knio.output_tables[0] = knio.Table.from_pandas(out)else:    # ========= 3) 全ペアのMcNemar 検定=========    rows = []    cols = X.columns.tolist()    n_subjects = X.shape[0]    for i in range(len(cols)):        for j in range(i+1, len(cols)):            c1, c2 = cols[i], cols[j]            s1 = X[c1].astype("float")            s2 = X[c2].astype("float")            # 欠損を除去（両列とも値がある行だけ）           mask = s1.notna() & s2.notna()            x = s1[mask].astype(int).to_numpy()            y = s2[mask].astype(int).to_numpy()            n = len(x)            # 2x2 集計           a = int(np.sum((x == 1) & (y == 1)))  # 11            b = int(np.sum((x == 1) & (y == 0)))  # 10            c = int(np.sum((x == 0) & (y == 1)))  # 01            d = int(np.sum((x == 0) & (y == 0)))  # 00            b_plus_c = b + c            # b+c=0 の場合、両者が完全一致→統計は0, p=1 と解釈（定義上は検定不能）           if b_plus_c == 0:                stat, pval = 0.0, 1.0            else:                # 大標本ではexact=False, continuity correctionありが一般的               table = np.array([[a, b], [c, d]], dtype=int)                res = mcnemar(table, exact=False, correction=True)                stat = float(res.statistic)                pval = float(res.pvalue)            # 効果量の目安（方向と規模を把握）：b - c) / (b + c)            # 正ならc1 が優位（1=1 & c2=0 が多い）、負ならc2 が優位           eff = np.nan if b_plus_c == 0 else (b - c) / b_plus_c            rows.append({                "model_A": c1,                "model_B": c2,                "n_used": n,                "a_11": a,                "b_10": b,                "c_01": c,                "d_00": d,                "b_plus_c": b_plus_c,                "mcnemar_stat": stat,                "p_raw": pval,                "effect_(b-c)/(b+c)": eff            })    res_df = pd.DataFrame(rows)    # ========= 4) 多重比較補正（olm 法）========    # Holm: p を昇順に並べ、_adj = max_{k<=i} ( (m-k+1) * p_(k) )    # 実装：標準的に逐次補正、単調性を保つ   m = len(res_df)    order = np.argsort(res_df["p_raw"].to_numpy())    p_sorted = res_df["p_raw"].to_numpy()[order]    holm_adj = np.empty_like(p_sorted, dtype=float)    for rank, p in enumerate(p_sorted, start=1):        holm_adj[rank-1] = (m - rank + 1) * p    # 単調非減少に調整   holm_adj = np.maximum.accumulate(holm_adj)    # 1 を超えないようクリップ   holm_adj = np.minimum(holm_adj, 1.0)    # 元の順へ戻す   p_adj_holm = np.empty_like(holm_adj)    p_adj_holm[order] = holm_adj    res_df["p_adj_holm"] = p_adj_holm    # 有意フラグ（例：%）   alpha = 0.05    res_df["significant_(holm_0.05)"] = res_df["p_adj_holm"] < alpha    # 並び替え（補正後p 値→生p 値）   res_df = res_df.sort_values(["p_adj_holm", "p_raw", "model_A", "model_B"], ascending=[True, True, True, True])    # ========= 5) 出力=========    knio.output_tables[0] = knio.Table.from_pandas(res_df)
