# Supplementary material for: Machine learning prediction of long-term sickness absence due to mental disorders using Brief Job Stress Questionnaire data
Source: Sci Rep. 2025 Dec 16;16:2908. doi: 10.1038/s41598-025-32857-3 (PMC12830388; doi:10.1038/s41598-025-32857-3)
Supplement: Supplementary file 2 — Supplementary Material 2 [file 41598_2025_32857_MOESM2_ESM.zip › Codes/borderline-SMOTE.py]

import pandas as pdfrom imblearn.over_sampling import BorderlineSMOTEimport knime.scripting.io as knio# --- KNIME から入力（ort0）--input_df = knio.input_tables[0].to_pandas()# 目的変数と特徴量（列名"MentalSL" は適宜変更） = input_df["SL"].astype(int)X = input_df.drop(columns=["SL"])# --- Borderline-SMOTE の実行---smote = BorderlineSMOTE(kind='borderline-1', random_state=42)X_res, y_res = smote.fit_resample(X, y)# --- 結果をまとめる---df_resampled = pd.DataFrame(X_res, columns=X.columns)df_resampled["SL"] = y_res# --- KNIME へ出力（ort0）--knio.output_tables[0] = knio.Table.from_pandas(df_resampled)
