# Supplementary material for: Machine learning prediction of long-term sickness absence due to mental disorders using Brief Job Stress Questionnaire data
Source: Sci Rep. 2025 Dec 16;16:2908. doi: 10.1038/s41598-025-32857-3 (PMC12830388; doi:10.1038/s41598-025-32857-3)
Supplement: Supplementary file 2 — Supplementary Material 2 [file 41598_2025_32857_MOESM2_ESM.zip › Codes/ADASYN.py]

# --- KNIME Python Script: ADASYN によるクラス不均衡補正（出力 1テーブル）---# 依存 imbalanced-learn (imblearn), scikit-learn, pandas, numpy# 入力 Input Table 0 に"MentalSL" を含む学習テーブル 出力 Output Table 0 にADASYN 後のサンプルmport numpy as npimport pandas as pdimport knime.scripting.io as kniofrom imblearn.over_sampling import ADASYN# ======= 設定=======TARGET_COL = "SL"       # 目的変数列名ANDOM     = 42N_NEIGHBORS = 5               # ADASYN のk 近傍AMPLING   = "auto"           # 生成比率 =====================# 入力f = knio.input_tables[0].to_pandas().copy()# チェックf TARGET_COL not in df.columns:    knio.output_tables[0] = knio.Table.from_pandas(        pd.DataFrame({"error":[f"目的変数列'{TARGET_COL}' が見つかりません。]})    )else:    # y とX を分割   y = pd.to_numeric(df[TARGET_COL], errors="coerce").astype("Int64")    X = df.drop(columns=[TARGET_COL])    # one-hot（DASYNは数値のみを想定）   X = pd.get_dummies(X, drop_first=False)    # 欠損補完（数値は平均）   num_cols = X.columns    if len(num_cols) == 0:        knio.output_tables[0] = knio.Table.from_pandas(            pd.DataFrame({"error":["特徴量がありません（ll NaN or 非数で削除された可能性）。]})        )    else:        X[num_cols] = X[num_cols].apply(pd.to_numeric, errors="coerce")        X[num_cols] = X[num_cols].fillna(X[num_cols].mean())        # y の欠損は落とす（と同じ行だけに揃える）       valid = y.notna()        X = X.loc[valid].reset_index(drop=True)        y = y.loc[valid].astype(int).reset_index(drop=True)        # ADASYN 実行       adas = ADASYN(random_state=RANDOM, n_neighbors=N_NEIGHBORS, sampling_strategy=SAMPLING)        X_res, y_res = adas.fit_resample(X, y)        # 出力テーブル作成（ターゲットは最後の列に）       out = pd.DataFrame(X_res, columns=X.columns)        out[TARGET_COL] = y_res.values        knio.output_tables[0] = knio.Table.from_pandas(out)
